# Histopathological Analysis of Lipopolysaccharide-Induced Liver Inflammation and Thrombus Formation in Mice: The Protective Effects of Aspirin

**DOI:** 10.3390/cimb46120856

**Published:** 2024-12-18

**Authors:** Hayate Saitoh, Miina Sakaguchi, Fumito Miruno, Naoto Muramatsu, Nozomi Ito, Kanako Tadokoro, Kiyoharu Kawakami, Kazuhiko Nakadate

**Affiliations:** Department of Functional Morphology, Meiji Pharmaceutical University, 2-522-1 Noshio, Kiyose 204-8588, Japan; y201111@std.my-pharm.ac.jp (H.S.); y201117@std.my-pharm.ac.jp (M.S.); y201290@std.my-pharm.ac.jp (F.M.); y201295@std.my-pharm.ac.jp (N.M.); s212009@std.my-pharm.ac.jp (N.I.); s212052@std.my-pharm.ac.jp (K.T.); k-kawakami@my-pharm.ac.jp (K.K.)

**Keywords:** lipopolysaccharide, thrombus, blood clot, liver, aspirin, inflammation, correlative light and electron microscopy, scanning electron microscopy

## Abstract

Hepatitis, a significant medical concern owing to its potential to cause acute and chronic liver disease, necessitates early intervention. In this study, we aimed to elucidate the histopathological features of lipopolysaccharide-induced hepatitis in mice, focusing on tissue alterations. The results demonstrated that hepatocytes exhibited decreased eosin staining, indicating cellular shrinkage, whereas sinusoids were swollen with blood cells. Detailed electron microscope analysis identified these blood cells as leukocytes and erythrocytes, which confirmed a thrombus formation within the liver. Pre-treatment with aspirin significantly attenuated these pathological changes, including reductions in inflammatory markers such as C-reactive protein, interleukin-1β, and tumor necrosis factor-alpha. These findings highlight aspirin’s anti-inflammatory and antiplatelet effects in mitigating liver inflammation and thrombus formation. In this study, we highlighted the potential of aspirin as a therapeutic agent for liver inflammation, in addition to providing insights into hepatocyte alterations and sinusoidal blood cell aggregation in liver inflammation. Aspirin, through the protection of endothelial cells and reduction of cytokine levels, may have broader applications in managing liver disease and other systemic inflammatory conditions. This emphasizes its value in prevention and therapy.

## 1. Introduction

Hepatitis is characterized by an inflammatory response in the liver that may progress to acute or chronic liver disease, cirrhosis, and hepatocellular carcinoma [1,2]. The liver is an important organ for detoxification, metabolism, and immune regulation. In addition, the progression of conditions associated with hepatitis can have serious health consequences [3]. The causes of hepatitis are diverse, including viruses, alcohol, drugs, and metabolic and immune abnormalities; in addition, lipopolysaccharide (LPS)-induced inflammatory responses have been highlighted as an important contributing factor [4,5,6]. This inflammatory response is known as hepatocellular injury [7], which leads to liver damage, hepatocyte injury, sinusoidal endothelial destruction, and thrombus (blood clotting) formation.

In the pathogenesis of LPS-induced hepatitis, sinusoidal and vascular endothelial cells in the liver are damaged, leading to abnormal activation and aggregation of platelets and the formation of thrombi in the blood vessels [8,9]. The histopathological changes in the liver following LPS administration include hepatocyte necrosis, inflammatory cell infiltration, and thrombus formation, which may significantly affect the progression and prognosis of hepatitis. These lesions may have a significant impact on the progression and prognosis of hepatitis.

Thrombosis occurs in atherosclerotic and chronic inflammatory diseases and causes serious complications, including stroke, myocardial infarction, and pulmonary thromboembolism [10,11,12]. These diseases are the leading causes of death worldwide, and their risk of development is significantly high in elderly and chronically ill patients [13]. In addition, recent studies have indicated that thrombosis occurs in organs such as the liver and intestine, suggesting that visceral thrombosis may worsen liver disease and intestinal ischemia [14,15]. In clinical practice, thrombosis prevention and management are important, and antiplatelet and anticoagulant drugs play a central role in the treatment [16,17].

Thrombosis is an important pathogenesis in cardiovascular and chronic inflammatory diseases involving several reactions, including vascular endothelial damage and platelet aggregation [18,19]. In the liver, damage to sinusoidal endothelial cells leads to impaired blood flow and abnormal platelet aggregation, which can lead to thrombus formation [14]. These reactions promote hepatic dysfunction and the progression of inflammatory liver disease [20].

LPS, a cell wall component of gram-negative bacteria, is an exogenous trigger that strongly induces an immune response, and strongly activates the immune response upon entry into the bloodstream, causing the release of inflammatory cytokines and chemokines [7,21,22]. The LPS-stimulated inflammatory response damages sinusoidal and vascular endothelial cells in the liver, leading to platelet activation and aggregation, and increasing the risk of thrombus formation [23,24]. The levels of inflammatory markers, including the acute phase protein, C-reactive protein (CRP), increase rapidly in response to LPS stimulation, which increases the risk of thrombus formation with an increase in systemic inflammation. CRP, an acute-phase protein produced by the liver, is significantly increased during inflammation and tissue injury and is an important marker reflecting the level of systemic inflammation, due to its marked increase during inflammation and tissue damage [25,26]. In addition, LPS-induced inflammatory response enhances the production of pro-inflammatory cytokines, including tumor necrosis factor (TNF-α) and interleukins (IL-1β), which further increases vascular permeability and platelet aggregation, thereby increasing the risk of thrombus formation [27,28,29].

Aspirin is widely used as a general anti-platelet agent and inhibits the formation of prostaglandins and thromboxane A2 (TXA2) primarily through the irreversible inhibition of cyclooxygenase (COX) [30,31]. TXA2 causes platelet aggregation and vasoconstriction, whereas aspirin prevents platelet aggregation and thrombus formation by inhibiting its production [32,33]. Furthermore, aspirin reportedly has anti-inflammatory and anti-platelet effects and contributes to the suppression of inflammatory markers, such as CRP, TNF-α, and IL-1β [34]. Therefore, aspirin has gained attention as an effective agent for reducing LPS-induced inflammatory responses and preventing thrombus formation [34]. The mechanism by which aspirin suppresses LPS-induced inflammatory responses is multifaceted and may reduce vascular endothelial damage through suppression of inflammatory markers including CRP and TNF-α. Following LPS administration, a rapid rise in CRP and increases in TNF-α and IL-1β are observed [35,36], before aspirin expectedly suppresses the elevation of these inflammatory markers and reduces the risk of thrombus formation. In addition, aspirin may reduce thrombus formation by reducing oxidative stress in endothelial cells and preventing increased vascular permeability [37]. These actions indicate aspirin’s potential as an anti-platelet agent and a part of thrombosis prevention through the suppression of systemic inflammation.

In this study, we aimed to analyze the histopathology of LPS-induced thrombus formation, especially in the liver, and the preventive effect of aspirin and its mechanism of action on inflammatory markers (CRP, TNF-α, and IL-1β).

## 2. Materials and Methods

### 2.1. Animals

We used 42 eight-week-old male ICR mice from Japan SLC in this study. All experimental procedures adhered to the guidelines in the National Institutes of Health Guide for the Care and Use of Laboratory Animals. The Laboratory Animal Ethics Committee of Meiji Pharmaceutical University approved the experiments (approval number: 2704, 1 April 2023 and 2024). Finally, we took measures to minimize animal suffering and the number of animals.

### 2.2. LPS Induction and Aspirin Administration

LPS was administered as previously described [38]. Twenty-three mice were administered LPS (Wako Pure Chemical Industries, Tokyo, Japan) intraperitoneally at a dose of 1 mg/kg body weight. An equal volume of saline was administered intraperitoneally to mice who did not develop hepatitis. The data of four animals from each group were included in the histopathological analysis.

Aspirin (Bayer Aspirin Tablets, Sato Pharmaceutical Co. Ltd., Tokyo. Japan) was administered orally to 15 animals, once daily at 2 mg/0.1 mL saline per kg body weight. The controls (15 animals) received the same dose of saline, and after 7 days, LPS or saline was administered to 12 animals.

### 2.3. Tissue Preparation, Histopathology, and Scanning Electron Microscope (SEM) Analysis

The tissues were prepared for histochemical and SEM analyses according to a previously described protocol [38,39,40]. Mice were deeply anesthetized 24 h after LPS (4 mice) or saline (4 mice), or LPS after 7 days of aspirin (4 mice) administration for histopathological analysis. The liver was removed and postfixed in the same fixative overnight at 4 °C.

Liver sections were prepared for hematoxylin and eosin (HE) staining analysis by sectioning the tissue to 5-mm-thick sections using a slicer. Subsequently, the sections were subjected to several procedures. The sections were initially exposed to increasing concentrations of ethanol, cleared with Lemosol A (Wako Pure Chemical Industries, Ltd., Japan), and finally embedded in paraffin. The paraffin-embedded liver blocks were sectioned into 5-μm thick slices using a sliding microtome (REM-710, Yamato Kohki Industrial, Tokyo, Japan). The sections were mounted on glass slides, deparaffinized using Lemosol A, and immersed in a gradient of ethanol and distilled water. Furthermore, they were subjected to HE staining, followed by dehydration using increasing ethanol concentrations. Finally, the sections were cleared using Lemosol A and covered with coverslips. The stained sections were captured using a CCD camera (BZ-X700, Keyence, Osaka, Japan).

The 5-mm-thick sections were cut for SEM observation. Following fixation with 1% osmium tetroxide (OsO_4)_ solution for 2 h, the sections were dehydrated and replaced with t-butyl alcohol solution. Subsequently, after lyophilization, the liver tissue was placed on an aluminum table for SEM (IT-800, JEOL, Tokyo. Japan).

### 2.4. Correlative Light and Electron Microscope Analysis

The paraffin-embedded liver blocks were sectioned into 5-μm-thick slices for correlative light and electron microscopy analyses. Sections were mounted on glass slides, deparaffinized using Lemosol A, and immersed in a gradient of ethanol and distilled water. The sections were stained with HE and washed severally with distilled water. The sections were coverslipped with distilled water, and images were captured using a CCD camera (BZ-X700, Keyence, Japan). Following photography, the cover glass was removed, and sections were dehydrated with ethanol, dried, and treated with an OsO_4_ coater. The field of view was observed using SEM and optical microscopy.

### 2.5. Measurement of CRP, IL-1β, and TNF-α in Blood

Mice serum CRP, IL-1β, and TNF-α concentrations were determined using an ELISA kit (ab157712, ab197742, ab208348, Abcam) following the manufacturer’s protocol. To examine the effect of pre-administered aspirin on each value, the aspirin and saline groups were compared. Aspirin was administered for 7 days, and whole blood samples from heart were taken after deep anesthesia without LPS (0H, 3 animals), 2 h (2H, 3 animals), 6 h (6H, 3 animals), 12 h (12H, 3 animals) and 24 h (24H, 3 animals) after LPS administration. Subjects were pre-treated with saline for 7 days, and whole blood samples were collected without LPS administration (0H, 3 animals), 2 h after LPS administration (2H, 3 animals), 6 h (6H, 3 animals), 12 h (12H, 3 animals), and 24 h (24H, 3 animals) after deep anesthesia.

Blood samples were centrifuged (1500 rpm, 10 min, 4 °C) to obtain serum, which was stored at −20 °C until analysis. Serum samples were brought to room temperature and diluted appropriately before analysis. The ELISA procedure was as follows: standard solutions and diluted serum samples were added to the ELISA plate and incubated at room temperature for 2 h. The wells were washed four times using a wash solution. Subsequently, the detection antibody was added, and samples were incubated for 10 min at room temperature. The substrate solution (TMB) was added after washing, and the reaction proceeded in the dark for 5 min. The reaction was stopped by the addition of stop solution and the absorbance was measured at 450 nm using a microplate reader. Standard curves were generated to calculate the CRP, IL-1β, and TNF-α concentrations in each sample. The measurements were repeated in triplicate for each sample, and the results are expressed as mean ± standard deviation.

### 2.6. Data Analysis

The investigators evaluated 200 distinct cells per mouse across the four mice in each group to assess the hepatocyte area. The number of hematoxylin-positive cells within the sinusoids was determined by examining 10 separate images per mouse, with four mice per group. Statistical analysis was conducted using analysis of variance and statistical significance set at *p* < 0.05.

## 3. Results

### 3.1. Histopathological Analysis

**Figure 1:**
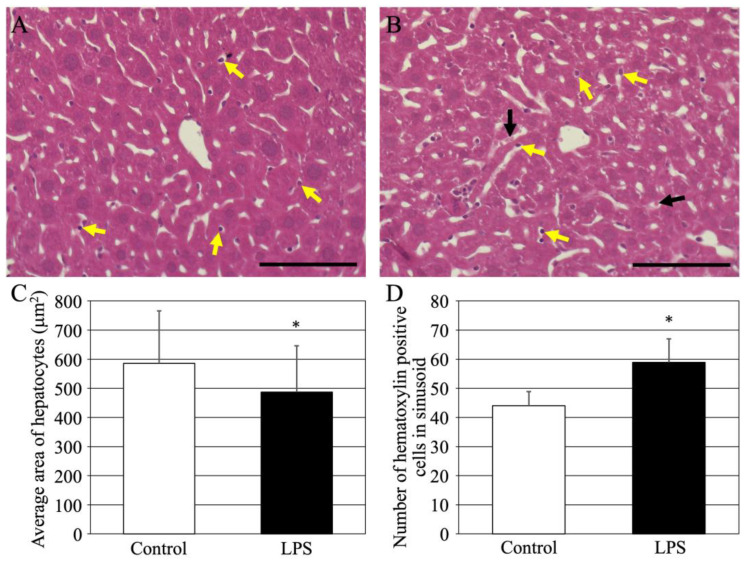
HE-stained images of livers from normal mice (**A**) and LPS-treated mice (**B**). Yellow arrows indicate hematoxylin-positive cells in the sinusoids. Black arrows indicate hematoxylin-negative cells within the sinusoids. Scale bars in (**A**,**B**) are 100 μm. (**C**) Average area in control (white bar) and LPS-treated mice (black bar) of hepatocytes (200 cells/animal, four animals from each group). (**D**) Number of hematoxylin-positive cells within sinusoids in control (white bar) and LPS-treated mice (black bar) of (numbers/200,000 μm^2^, 10 pictures/animal, four animals from each group). Each graph shows the mean ± standard deviation. *; *p* < 0.05 compared to control value. HE, hematoxylin-eosin; LPS, liposaccharide. All sale bars are 100 μm.

First, acute LPS-induced hepatitis was analyzed histopathologically. Mice were injected with saline or LPS, and after 24 h, their livers were removed, and HE-stained specimens were analyzed (Figure 1). The livers of the controls (normal) were healthy (Figure 1A). However, hepatocytes in the hepatitis-induced liver (Figure 1B) showed reduced eosin staining. The area of hepatocytes was quantified because the sinusoids appeared to be vastly enlarged (Figure 1C). The results revealed significantly shrunken hepatocytes in mice with LPS-induced liver hepatitis. In addition, a large number of hematoxylin-positive cells were found in the sinusoids of mice with LPS-induced liver hepatitis (yellow arrows in Figure 1A,B). Quantitative results showed a significant increase in the number of hematoxylin-positive cells (Figure 1D). In contrast, there was an increasing trend in the number of hematoxylin-negative cells (black arrows in Figure 1B) in the sinusoids of mice with LPS-induced liver hepatitis; however, the increase was not significant.

### 3.2. Correlative Light and Electron Microscopy Analysis

**Figure 2:**
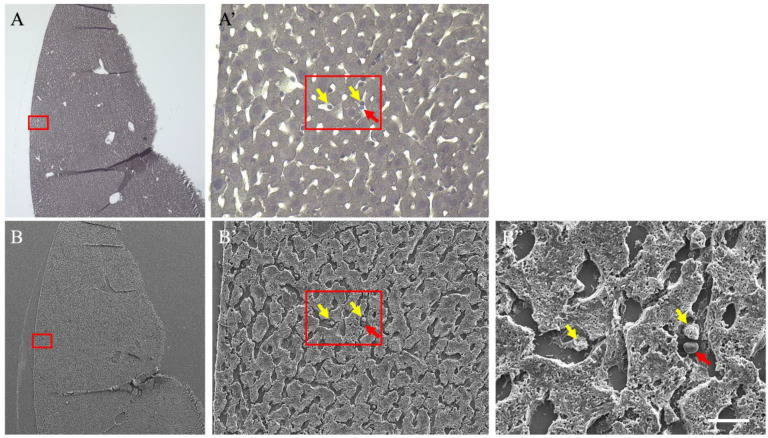
Correlative light and electron microscopical (CLEM) images of livers from liposaccharide-treated mice. (**A**,**A’**) Hematoxylin-stained images. (**B**–**B”**) Scanning electron microscope images of the same tissue. (**A’**) is a magnified image of the red frame of A. (**B’**) is a magnified image of the red frame of (**B**). (**B”**) is a magnified image of the red frame of (**B’**). Yellow arrows indicate hematoxylin-positive cells in the sinusoids. Red arrows indicate hematoxylin-negative cells. The scale bar in (**B”**) is 10 μm.

A correlative light and electron microscopic method was used for detailed analysis to clarify the hematoxylin-positive cells observed in the sinusoids (Figure 2). HE staining, used in histopathological analysis, as shown in Figure 1, was not performed rather only hematoxylin staining was conducted (Figure 2A,A’). Hematoxylin-positive cells were identified under an optical microscope, treated with an osmium coater, and observed in the same field of view using a SEM (Figure 2B–B”). The hematoxylin-positive cells (yellow arrows) identified under an optical microscope were observed under an SEM. They were recognized as leukocytes using their morphology. In contrast, hematoxylin-negative cells (red arrows) were recognized as erythrocytes based on their morphology. Further analyses of these cells are difficult to perform using SEM, which can observe the cell surface; however, these analyses indicate that LPS treatment induces a significant accumulation of leukocytes in the sinusoids of the liver, and the aggregation of erythrocytes.

### 3.3. Histopathological and SEM Analysis of the Blood Vessels in the Liver

**Figure 3:**
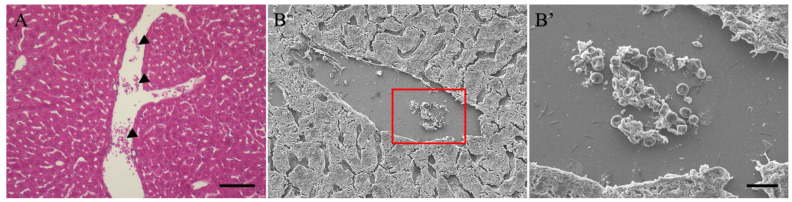
HE-stained (**A**) and SEM (**B**,**B’**) images of liver from LPS-treated mice. (**B’**) is a magnified image of the red frame of (**B**). Black arrowheads indicate hematocytes in the blood vessel. The scale bar in (**A**) is 10 μm, in (**B’**) is 10 μm. HE, hematoxylin-eosin; LPS, liposaccharide; SEM, scanning electron microscope.

The above correlative light and electron microscopical analysis indicated that blood accumulation (aggregation) may occur in the sinusoids; however, histopathological analysis was performed on the blood vessels present in the liver (Figure 3). HE-stained images of livers with acute LPS-induced hepatitis showed many blood cells remaining in the blood vessels (Figure 3A). We observed the morphology of the accumulated cells using SEM Figure 3B,B’ based on the staining characteristics, as they may accumulate alongside erythrocytes and leukocytes, and possibly thrombi. Morphological analysis revealed that the accumulated cells were mostly erythrocytes, composed of leukocytes and platelets.

### 3.4. SEM Analysis of the Blood Vessels and Hepatocytes in the Liver

**Figure 4:**
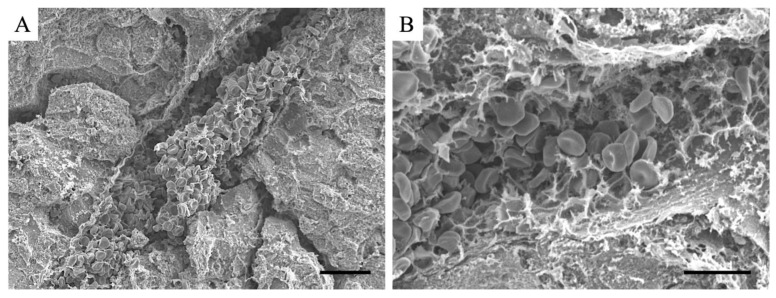
SEM images of livers from LPS-treated mice. (**A**) lower magnified image and (**B**) higher magnified image. The scale bar in (**A**) is 20 μm, (**B**) is 10 μm. LPS, liposaccharide; SEM, scanning electron microscope.

In 5-μm-thick sections, an accumulation of blood cells was observed in blood vessels in the liver in LPS-induced liver hepatitis; however, it was impossible to determine if the accumulation was a thrombus. Therefore, SEM analysis was performed on thick sections (Figure 4). We identified numerous thrombi in the vessels of patients with LPS-induced liver hepatitis. These results indicate that thrombi are induced in blood vessels within the liver in LPS-induced liver hepatitis; however, they could not be determined in thin sections.

Based on these histopathological analyses and a series of SEM analyses, the accumulation of blood cells in the blood vessels observed under an optical microscope was determined to be a thrombus. Therefore, the presence or absence of a thrombus can be determined by the presence or absence of blood cell accumulation under an optical microscope.

**Figure 5:**
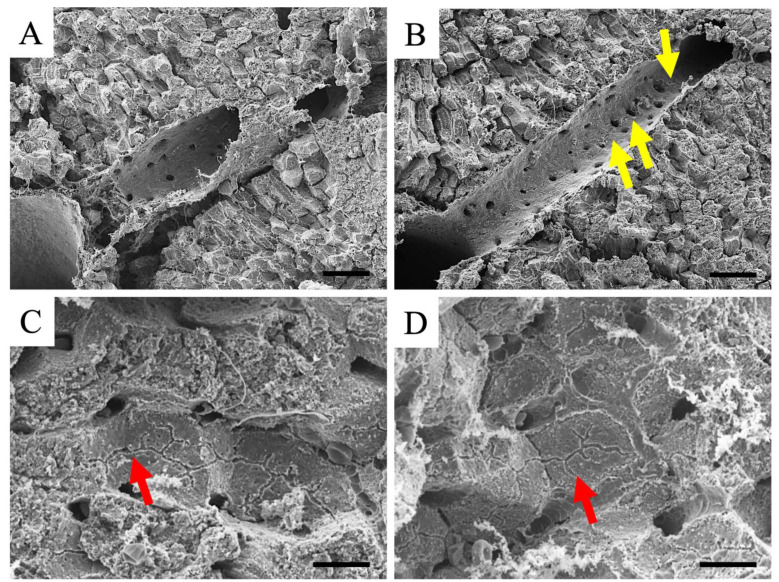
SEM images of livers from normal (**A**,**C**) and LPS-treated (**B**,**D**) mice. (**A**,**B**) The images of the inner surface of endothelial cells. (**C**,**D**) The images of capillary bile ducts. Yellow arrows in B indicate the rough inner surfaces of endothelial cells. Red arrows in (**C**,**D**) indicate the capillary bile ducts. LPS, liposaccharide; SEM, scanning electron microscope. Scale bars in A and B are 50 μm, and in C and D are 10 μm.

Using SEM, a detailed observation of the inner surface of the vessel and the surface of the hepatocytes was possible. Therefore, we observed the inner surface of the blood vessels, in which no thrombus was observed, for confirmation (Figure 5A,B). The inner surface of endothelial cells within the liver of the controls was healthy (Figure 5A). In contrast, in the blood vessels within the liver in LPS-induced liver hepatitis, some areas showed rough inner surfaces (yellow arrows in Figure 5B), whereas others showed intact surfaces. A large thrombus was not noticed in the observed areas of the endothelium in this analysis; however, it is highly possible that the thrombus was caused by the disruption of the endothelium. Furthermore, capillary bile ducts were observed on the surface of the hepatocytes (Figure 5C,D). Using this analysis, no significant difference was observed in the capillary bile ducts of LPS-induced liver hepatitis (red arrow in Figure 5D) compared to the controls (red arrow in Figure 5C). This result indicates a normal bile transport function in the hepatocytes of LPS-induced liver hepatitis mice.

### 3.5. Thromboprophylaxis Effect of Aspirin Pre-Administration

**Figure 6:**
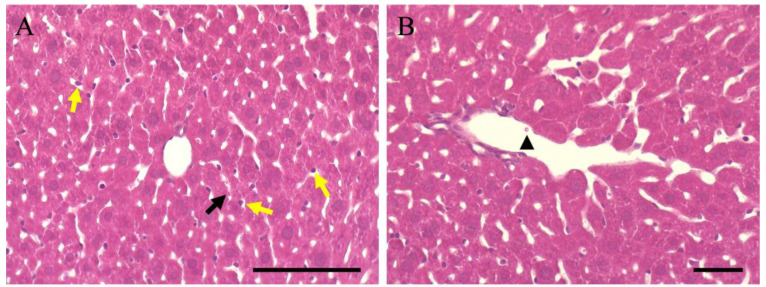
HE-stained image of hepatitis-induced mouse liver by LPS after pre-administration of aspirin. Yellow arrows indicate hematoxylin-positive cells in the sinusoids. Black arrows indicate hematoxylin-negative cells within the sinusoids. Black arrowheads indicate hematocytes in the blood vessel. Scale bars in (**A**,**B**) are 100 μm. HE, hematoxylin-eosin; LPS, liposaccharide; SEM.

Aspirin is believed to have a prophylactic effect on blood clots; however, there are no reports on its preventive effects on hepatocytes and liver thrombosis. Therefore, aspirin was administered for one week, followed by LPS-induced inflammation. Histopathological analysis showed that the size (area) of hepatocytes was 560.28 ± 167.2 (Figure 6A), which was not significantly different from that of normal animals (586.05 ± 180.1; Figure 1C) and improved. In addition, the number of hematoxylin-positive cells in the sinusoids was 47.12 ± 6.5 (Figure 6A), which was not significantly different from that in normal animals (44.19 ± 4.9; Figure 1D), indicating improvement. However, eosin staining of hepatocytes showed a slightly lighter tone, suggesting that the effect of LPS was reflected in the hepatocytes. In contrast, no accumulation of blood cells, considered a thrombus, was observed in the blood vessels of the liver. This indicates that aspirin is effective on the inflammation induced by LPS in the liver; however, its preventive effect on hepatocytes may not be complete.

**Figure 7:**
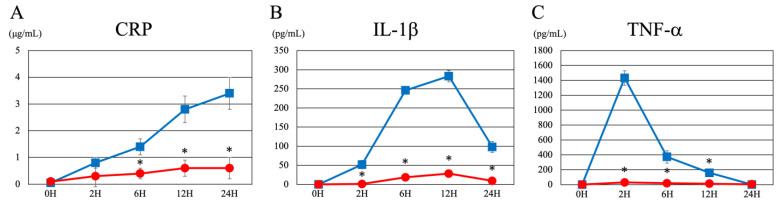
Changes in each inflammatory marker over time after saline pre-treatment and aspirin pre-treatment. (**A**) CRP (**B**) IL-1β (**C**) TNF-α. The blue line represents the control group (saline pre-treatment). The red line represents the aspirin group. 0H indicates values without LPS. 2H, 6H, 12H, and 24H indicate values 2 h, 6 h, 12 h, and 24 h after LPS administration, respectively. Each graph shows the mean ± standard deviation. *; *p* < 0.05 compared to control value. CRP, C-reactive protein; IL-1β, interleukin 1β; TNF-α; tumor necrosis factor alpha; LPS, liposaccharide.

Next, we examined whether aspirin’s reduction of liver inflammation was effective in preventing inflammation in the blood (Figure 7). First, CRP levels increased with time after LPS administration; however, they were significantly reduced in the pre-aspirin group (Figure 7A). IL-1b levels peaked at 6 to 12 h after LPS administration; nevertheless, they were significantly reduced by pre-aspirin administration (Figure 7B). TNF-a peaked at 2 h after LPS administration; however, this increase was significantly reduced by pre-administration of aspirin (Figure 7C). These findings suggest that aspirin pre-administration significantly decreases liver and blood inflammation.

## 4. Discussion

In this study, we investigated LPS-induced histopathological changes in the liver and the preventive effect of aspirin. We investigated the effects of LPS on hepatocytes, vascular endothelial damage, thrombus formation, and inflammatory cell infiltration. Hepatocyte shrinkage, sinusoidal swelling, and vascular endothelial damage were observed after LPS administration (Figure 1). Histopathological analysis revealed abnormal accumulation of leukocytes and erythrocytes in the sinusoids and blood vessels of the liver. Electron microscopy demonstrated that this accumulation was responsible for inflammation, induction of platelet aggregation, and thrombus formation, suggesting its major role in the progression of inflammation (Figure 2 and Figure 3).

Furthermore, we discovered that the pre-administration of aspirin before LPS administration alleviated these pathological changes (Figure 6). In addition, aspirin reduced the levels of LPS-induced inflammatory markers (CRP, IL-1β, and TNF-α) and had an inhibitory effect on vascular endothelial damage and platelet aggregation (Figure 7). These results confirmed that the pre-administration of aspirin inhibited thrombus formation and the abnormal accumulation of inflammatory cells in the liver, effectively blocking the progression of LPS-induced liver inflammation. These findings suggested that aspirin’s anti-inflammatory and antiplatelet effects may be useful as prophylaxis against LPS-induced liver injury.

This study is consistent with previous findings on LPS-induced liver inflammation, confirming that LPS produces a potent inflammatory response that causes the elevation of CRP, IL-1β, and TNF-α [41,42]. This demonstrated the process of vascular endothelial damage within the liver, the promotion of platelet aggregation, and the occurrence of thrombus formation. These inflammatory cascades occur when LPS invades the bloodstream, thereby stimulating the immune system and the production of multiple cytokines and chemokines, leading to endothelial cell damage and platelet activation [7,21,22]. We believe that these findings are consistent with the immune response mechanisms triggered when LPS functions as a cell wall component in gram-negative bacteria, and provide histopathological evidence for a previous study [22].

In addition, we gained a new perspective on the effects of LPS on the liver, particularly in the sinusoids. In this study, we demonstrated that LPS-induced liver tissue damage is accompanied by abnormal histopathological changes, including leukocyte and erythrocyte accumulation and endothelial roughness. The accumulation of blood cells in the sinusoids can cause thrombus formation and inflammatory cell infiltration in the blood vessels. Our results indicate that the release of pro-inflammatory cytokines is important in the primary mechanism by which LPS causes endothelial damage in the liver, and is consistent with previous studies [22,42,43]. In addition, pathological findings, including endothelial cell roughness and abnormal blood cell accumulation, are important indicators of LPS-induced microvascular abnormalities within the liver. Furthermore, they provide valuable information for understanding the progression of liver inflammation which causes vascular system damage.

In this study, we investigated the mechanism by which aspirin reduces LPS-induced inflammatory damage. Aspirin acts primarily by inhibiting TXA2 formation through inhibition of COX enzymes, particularly COX-1 and COX-2 [30,31,32,33]. TXA2 is an important mediator of platelet aggregation, aspirin prevents platelet aggregation and blood clot formation by suppressing its formation. This anti-platelet effect is expected to help maintain smooth blood flow and inhibit the accumulation of microthrombi in the liver’s blood vessels. Furthermore, aspirin has potent anti-inflammatory effects that may suppress LPS-induced hepatic inflammation. The anti-inflammatory effects of aspirin are associated with the inhibition of inflammatory markers such as CRP, IL-1β, and TNF-α. These cytokines play important roles in the inflammatory cascade, causing endothelial damage and thrombus formation [34]. The results of this study indicate that aspirin suppresses the production of these inflammatory mediators, thereby protecting the liver endothelial cells from damage and inhibiting thrombus formation.

More interestingly, aspirin may reduce oxidative stress through its antioxidant properties [44]. Inflammatory reactions induced by LPS have been shown to increase oxidative stress and damage endothelial cells; however, aspirin may promote endothelial cell protection by reducing the level of oxidative stress. In addition, aspirin has been reported to inhibit vascular permeability, which may reduce excessive fluid leakage from the blood vessels and tissue swelling [45]. These mechanisms of action may be important for reducing liver inflammation, protecting endothelial integrity, and ameliorating histopathological changes. Collectively, these results indicate that aspirin’s anti-platelet, anti-inflammatory, and antioxidant properties, may work together to reduce LPS-induced liver injury and contribute to tissue protection. This study suggests that aspirin is not just an anti-platelet agent, but has a multifaceted preventive and protective effects on liver inflammation.

This study’s findings indicate that aspirin may be useful as an adjunct therapy to reduce LPS-induced liver injury. Pre-administration of aspirin significantly reduced inflammatory markers (CRP, IL-1β, TNF-α, etc.) and thrombus formation, suggesting its potential in reducing the effects of systemic inflammation on the liver. In particular, aspirin use may play an important role in protecting the liver and preventing vascular complications such as sepsis and acute liver failure, where systemic inflammation is amplified through LPS. The effects of aspirin extend beyond its antiplatelet action; aspirin promotes the reduction of vascular permeability and protection of endothelial cells within the liver through its anti-inflammatory effects and reduction of oxidative stress. This multifaceted effect may have great clinical significance in the management of liver inflammation and the prevention of thrombosis, especially in patients prone to severe LPS-induced inflammatory liver injury. Furthermore, because LPS-induced endothelial damage and vascular inflammation can cause serious thrombotic events such as myocardial infarction and pulmonary thromboembolism, aspirin use may have a dual benefit for patients, by simultaneously inhibiting platelet aggregation and inflammation. Prophylactic aspirin administration in patients with chronic liver disease and high-risk groups should be considered. Patients with chronic liver disease often have coexisting abnormalities in their coagulation and inflammatory systems, and the risk of thrombus formation increases with worsening liver function. In the context of preventive medicine and long-term disease management, a comprehensive treatment strategy that includes aspirin should be considered, as it may reduce the risk of thrombosis in these patients and help maintain liver function.

This study had some limitations. First, an animal model, which may not fully replicate the LPS-induced inflammatory response in humans, was used. In addition, aspirin significantly reduced inflammatory marker levels; however, residual hepatocellular damage was observed, suggesting that its protective effect may not be complete at high doses of LPS. Therefore, future studies should investigate the long-term effects of aspirin on liver tissue repair and its combined effects with other anti-inflammatory drugs at different stages of liver inflammation. In addition, optimizing aspirin dosing schedules and examining its effects on other inflammatory pathways may suggest the possibility of enhancing therapeutic efficacy.

In this study, we demonstrated LPS-induced liver inflammation and thrombus formation through histopathological and micro-morphological examinations using electron microscopy. These results indicated that the accumulation of blood cells observed in specimens used in general pathology was likely a thrombus, and may provide useful information for clinical diagnosis. Furthermore, we discovered that aspirin pre-administration effectively reduced LPS-induced liver inflammation and thrombus formation, and exerted a protective effect on the liver through its antiplatelet and anti-inflammatory effects. These results indicated that aspirin contributed to the maintenance of liver health by suppressing inflammatory cytokines, protecting endothelial cells, and preventing platelet aggregation. In addition, aspirin can be applied as a therapeutic option in the liver and systemic inflammatory diseases. It is important to review its position in prevention and treatment strategies, including its cost-effectiveness and versatility. Further clinical studies are required to investigate the range of applications and optimal administration of aspirin, thereby contributing to the establishment of a comprehensive treatment for inflammatory diseases and thrombosis.

## 5. Conclusions

In this study, we elucidated LPS-induced liver inflammation and thrombus formation through histopathological and electron microscopy and micromorphological examinations, revealing that the accumulation of blood cells observed on routine pathology examination could be a thrombus. Furthermore, aspirin pre-administration effectively inhibited LPS-induced hepatitis and thrombus formation and conferred hepatoprotection through its antiplatelet and anti-inflammatory effects. Notably, aspirin inhibited inflammatory cytokines, protected endothelial cells, and prevented platelet aggregation, indicating its potential as a therapeutic agent for hepatic and systemic inflammatory diseases. Future clinical studies are expected to contribute to the establishment of a comprehensive treatment for inflammatory diseases and thrombosis by investigating the range of applications and optimal aspirin administration methods.

## Data Availability

The datasets used and/or analyzed during this study are available from the corresponding author upon reasonable request.

## References

[1] Ohtsuki T., Kimura K., Tokunaga Y., Tsukiyama-Kohara K., Tateno C., Hayashi Y., Hishima T., Kohara M. (2016). M2 Macrophages Play Critical Roles in Progression of Inflammatory Liver Disease in Hepatitis C Virus Transgenic Mice. J. Virol..

[2] Mohan P., Chandra R.S., Escolar D.M., Luban N.L. (2001). Inflammatory myopathy and hepatitis C in a pediatric patient: Role of liver biopsy in evaluating the severity of liver disease. Hepatology.

[3] Yoshihara S., Harada K., Ohta S. (2000). Metabolism of 1-methyl-4-phenyl-1,2,3,6-tetrahydropyridine (MPTP) in perfused rat liver: Involvement of hepatic aldehyde oxidase as a detoxification enzyme. Drug Metab. Dispos..

[4] Shin M.S., Kang E.H., Lee Y.I. (2005). A flavonoid from medicinal plants blocks hepatitis B virus-e antigen secretion in HBV-infected hepatocytes. Antiviral Res..

[5] Hayashi M., Kanda T., Nakamura M., Miyamura T., Yasui S., Nakamoto S., Wu S., Arai M., Imazeki F., Yokosuka O. (2014). Acute liver injury in a patient with alcohol dependence: A case resembling autoimmune hepatitis or drug-induced liver injury. Case Rep. Gastroenterol..

[6] Odena G., Chen J., Lozano J.J., Altamirano J., Rodrigo-Torres D., Affo S., Morales-Ibanez O., Matsushita H., Zou J., Dumitru R. (2016). LPS-TLR4 Pathway Mediates Ductular Cell Expansion in Alcoholic Hepatitis. Sci. Rep..

[7] Maldonado R.F., Sa-Correia I., Valvano M.A. (2016). Lipopolysaccharide modification in Gram-negative bacteria during chronic infection. FEMS Microbiol. Rev..

[8] Li M., Zhao Y., Liu X., Dang Z., Wang X., Jiang Y., Yang Z. (2019). Association and interaction between model for end-stage liver disease score and minimally invasive treatment with regard to mortality of patients with hepatitis B virus-associated hepatocellular carcinoma and portal vein tumor thrombi. Oncol. Lett..

[9] Kohler J., Ehler J., Kreikemeyer B., Bajorath R., Schurholz T., Oehmcke-Hecht S. (2020). The synthetic LPS binding peptide 19-2.5 interferes with clotting and prevents degradation of high molecular weight kininogen in plasma. Sci. Rep..

[10] Schafer A.I., Mann D.L. (2024). Thrombotic, Cardiovascular, and Microvascular Complications of Myeloproliferative Neoplasms and Clonal Hematopoiesis (CHIP): A Narrative Review. J. Clin. Med..

[11] Feigin V.L., Abate M.D., Abate Y.H., Abd ElHafeez S., Abd-Allah F., Abdelalim A., Abdelkader A., Abdelmasseh M., Abd-Elsalam S., Abdi P. (2024). Global, regional, and national burden of stroke and its risk factors, 1990–2021: A systematic analysis for the Global Burden of Disease Study 2021. Lancet Neurol..

[12] Gao X., Chen H., Huang Z., Lin J., Huang J., Chen Q. (2024). Correlation Between Neutrophil-to-Lymphocyte Ratio and Platelet-to-Lymphocyte Ratio with Risk Stratification Indicators and Thrombus Burden in Patients with Moderate-to-High Risk Acute Pulmonary Embolism, and Changes After Treatment. Clin. Appl. Thromb. Hemost..

[13] Qiu J., Yu Y., Wang Z., Hong L., Shao L., Wu J. (2024). Developing Individualized Follow-Up Strategies Based on High-Risk Recurrence Factors and Dynamic Risk Assessment for Locally Advanced Rectal Cancer. Cancer Med..

[14] Chen Z.H., Zhang X.P., Feng S., Feng J.K., Chai Z.T., Guo W.X., Shi J., Lau W.Y., Meng Y., Cheng S.Q. (2021). Liver resection versus intensity-modulated radiation therapy for treatment of hepatocellular carcinoma with hepatic vein tumor thrombus: A propensity score matching analysis. Hepatobiliary Surg. Nutr..

[15] Ichida A., Kokudo T., Shimada S., Hatano E., Kubo S., Kato Y., Ishikawa Y., Mori A., Baba H., Matsuyama Y. (2023). Liver Resection for Hepatocellular Carcinoma With Tumor Thrombus in the Inferior Vena Cava or Right Atrium: A Large-scale Multicenter Survey Conducted in Japan. Ann. Surg..

[16] Bultink I.E.M. (2024). Dialogue: Antiplatelet effects of hydroxychloroquine in patients with systemic lupus erythematosus evaluated by the total thrombus-formation analysis system (T-TAS). Lupus Sci. Med..

[17] Tourdot B.E., Stoveken H., Trumbo D., Yeung J., Kanthi Y., Edelstein L.C., Bray P.F., Tall G.G., Holinstat M. (2018). Genetic Variant in Human PAR (Protease-Activated Receptor) 4 Enhances Thrombus Formation Resulting in Resistance to Antiplatelet Therapeutics. Arterioscler. Thromb. Vasc. Biol..

[18] Kellermair L., Hofer C., Zeller M.W.G., Kubasta C., Bandke D., Weis S., Kellermair J., Forstner T., Helbok R., Vosko M.R. (2024). Endothelial receptor proteins in acute venous thrombosis and delayed thrombus resolution in cerebral sinus vein thrombosis. J. Neurol..

[19] Rossi E., Pericacho M., Kauskot A., Gamella-Pozuelo L., Reboul E., Leuci A., Egido-Turrion C., El Hamaoui D., Marchelli A., Fernandez F.J. (2023). Soluble endoglin reduces thrombus formation and platelet aggregation via interaction with alphaIIbbeta3 integrin. J. Thromb. Haemost..

[20] Ma J., Zhu J., Ding T., Cai L., Zhou C., Zhang Y. (2023). Thrombus formation in the suprahepatic inferior vena cava after microwave ablation in patients with hepatic metastasis: A case report. Thromb. J..

[21] Zhang J., Oueslati R., Cheng C., Zhao L., Chen J., Almeida R., Wu J. (2018). Rapid, highly sensitive detection of Gram-negative bacteria with lipopolysaccharide based disposable aptasensor. Biosens. Bioelectron..

[22] Gonzalez-Fernandez C., Bringas E., Oostenbrink C., Ortiz I. (2022). In silico investigation and surmounting of Lipopolysaccharide barrier in Gram-Negative Bacteria: How far has molecular dynamics Come?. Comput. Struct. Biotechnol. J..

[23] Hsu C.S., Chang S.H., Yang R.C., Lee C.H., Lee M.S., Kao J.K., Shieh J.J. (2024). Lipopolysaccharide-Induced Lysosomal Cell Death Through Reactive Oxygen Species in Rat Liver Cell Clone 9. Environ. Toxicol..

[24] Guo L., Liu Y., Chen Y., Xu J., Liu Y. (2023). Liver macrophages show an immunotolerance phenotype in nonalcoholic fatty liver combined with Porphyromonas gingivalis-lipopolysaccharide infection. Hua Xi Kou Qiang Yi Xue Za Zhi.

[25] Tiirola T., Sinisalo J., Nieminen M.S., Silvennoinen-Kassinen S., Paldanius M., Saikku P., Jauhiainen M., Leinonen M. (2007). Chlamydial lipopolysaccharide is present in serum during acute coronary syndrome and correlates with CRP levels. Atherosclerosis.

[26] Tenforde M.W., Gupte N., Dowdy D.W., Asmuth D.M., Balagopal A., Pollard R.B., Sugandhavesa P., Lama J.R., Pillay S., Cardoso S.W. (2015). C-reactive protein (CRP), interferon gamma-inducible protein 10 (IP-10), and lipopolysaccharide (LPS) are associated with risk of tuberculosis after initiation of antiretroviral therapy in resource-limited settings. PLoS ONE.

[27] Yang Y., Wang Y., Wang C., Wu S., Yao D. (2023). Macrophages and derived-TNF-alpha promote lipopolysaccharide-induced upregulation of endogenous beta-glucuronidase in the epithelial cells of the bile duct: A possible facilitator of hepatolithiasis formation. Clin. Res. Hepatol. Gastroenterol..

[28] Komegae E.N., Fonseca M.T., Steiner A.A. (2020). Diet-induced obesity attenuates the hypothermic response to lipopolysaccharide independently of TNF-alpha production. Temperature.

[29] Lee Y., Ju X., Cui J., Zhang T., Hong B., Kim Y.H., Ko Y., Park J., Choi C.H., Heo J.Y. (2024). Mitochondrial dysfunction precedes hippocampal IL-1beta transcription and cognitive impairments after low-dose lipopolysaccharide injection in aged mice. Heliyon.

[30] Tacconelli S., Contursi A., Falcone L., Mucci M., D’Agostino I., Fullone R., Sacco A., Zucchelli M., Bruno A., Ballerini P. (2020). Characterization of cyclooxygenase-2 acetylation and prostanoid inhibition by aspirin in cellular systems. Biochem. Pharmacol..

[31] Bastaki S.M.A., Padol I.T., Amir N., Hunt R.H. (2018). Effect of Aspirin and ibuprofen either alone or in combination on gastric mucosa and bleeding time and on serum prostaglandin E(2) and thromboxane A(2) levels in the anaesthetized rats in vivo. Mol. Cell Biochem..

[32] Yin Y., Geng H., Cui S., Hua-Ji L. (2022). Correlation of platelet-derived growth factor and thromboxane A2 expression with platelet parameters and coagulation indices in chronic altitude sickness patients. Exp. Physiol..

[33] Mizurini D.M., Aslan J.S., Gomes T., Ma D., Francischetti I.M., Monteiro R.Q. (2015). Salivary Thromboxane A2-Binding Proteins from Triatomine Vectors of Chagas Disease Inhibit Platelet-Mediated Neutrophil Extracellular Traps (NETs) Formation and Arterial Thrombosis. PLoS Negl. Trop. Dis..

[34] Feng Y., Zhang H., Dai S., Li X. (2024). Aspirin treatment for unruptured intracranial aneurysms: Focusing on its anti-inflammatory role. Heliyon.

[35] Li M., Wang J., Wang X., Li G. (2020). Clinical efficacy of aspirin combined with clopidogrel in treating cerebral infarction and its effect on serum hs-CRP, sICAM-1 and TNF-alpha. Exp. Ther. Med..

[36] Wu Z., Wu Y., Zhong W., Zhong Q., Rao S., Yu D., Luo X., Qiu F., Song Z., Jin D. (2023). The hepatoprotective effect of aspirin on carbon tetrachloride-induced hepatic fibrosis via inhibition of TGFbeta-1 pathway and pro-inflammatory cytokines IL-1beta and COX-2 in rats. Exp. Ther. Med..

[37] Rood K.M., Patel N., DeVengencie I.M., Quinn J.P., Gowdy K.M., Costantine M.M., Kniss D.A. (2023). Aspirin modulates production of pro-inflammatory and pro-resolving mediators in endothelial cells. PLoS ONE.

[38] Hikita M., Motojima K., Kamata S., Yoshida T., Tanaka-Nakadate S., Nakadate K. (2016). Protective Efficacy of the Ingestion of Mandarin Orange Containing beta-Cryptoxanthin on Lipopolysaccharide-induced Acute Nephritis. Yakugaku Zasshi.

[39] Itoh M., Nakadate K., Horibata Y., Matsusaka T., Xu J., Hunziker W., Sugimoto H. (2014). The structural and functional organization of the podocyte filtration slits is regulated by Tjp1/ZO-1. PLoS ONE.

[40] Nakadate K., Sono C., Mita H., Itakura Y., Kawakami K. (2023). Severe Acute Liver Dysfunction Induces Delayed Hepatocyte Swelling and Cytoplasmic Vacuolization, and Delayed Cortical Neuronal Cell Death. Int. J. Mol. Sci..

[41] Huang H., Wu S., Yan Y. (2023). Growth differentiation factor 11 suppresses intrahepatic inflammation via restricting NLRP3 inflammasome activation in LPS-induced liver injury. Cell Mol. Biol..

[42] Khan H.U., Aamir K., Jusuf P.R., Sethi G., Sisinthy S.P., Ghildyal R., Arya A. (2021). Lauric acid ameliorates lipopolysaccharide (LPS)-induced liver inflammation by mediating TLR4/MyD88 pathway in Sprague Dawley (SD) rats. Life Sci..

[43] Sae-Tan S., Kumrungsee T., Yanaka N. (2020). Mungbean seed coat water extract inhibits inflammation in LPS-induced acute liver injury mice and LPS-stimulated RAW 246.7 macrophages via the inhibition of TAK1/IkappaBalpha/NF-kappaB. J. Food Sci. Technol..

[44] Liang H., Shi H., Li Y., Wang D., Zhang Y. (2023). Mechanism of Aspirin oxidative stress regulating interleukin-induced apoptosis in nucleus pulposus cells in a rat model of intervertebral disc degeneration. Ann. Transl. Med..

[45] Serhan C.N., Takano T., Chiang N., Gronert K., Clish C.B. (2000). Formation of endogenous "antiinflammatory" lipid mediators by transcellular biosynthesis. Lipoxins and aspirin-triggered lipoxins inhibit neutrophil recruitment and vascular permeability. Am. J. Respir. Crit. Care Med..

